# Surgical strategies of complicated pheochromocytomas/paragangliomas and literature review

**DOI:** 10.3389/fendo.2023.1129622

**Published:** 2023-04-21

**Authors:** Xu Wang, Yang Zhao, Zhangcheng Liao, Yushi Zhang

**Affiliations:** Department of Urology, Peking Union Medical College Hospital, Chinese Academy of Medical Sciences and Peking Union Medical College, Beijing, China

**Keywords:** pheochromocytomas, paragangliomas, catecholamine, surgery, multidisciplinary treatment

## Abstract

Pheochromocytomas (PCC)/paragangliomas (PGL) are catecholamine (CA) -secreting neuroendocrine tumors, which are known as PPGL due to their histological and pathophysiological similarities. In addition to the typical triad of paroxysmal headache, palpitation, and sweating, PPGL may also be accompanied by symptoms and signs involving multiple organs and systems such as the cardiovascular system, digestive system, endocrine system, and nervous system. Currently, surgical resection is the first choice for PPGL. Safe and effective surgical management of complicated PPGL is the goal of clinical work. In this paper, we discuss this hot issue based on complicated PPGL cases, aiming to share our experience of the surgical management strategy of PPGL.

## 1 Introduction

Pheochromocytomas arise from chromaffin cells, which can produce CAs in the adrenal medulla, whereas paragangliomas arise from the extra-adrenal region (1). Sympathetic paragangliomas can produce CAs, which are generally distributed in paravertebral and pelvic areas, while non -CA-secreting-parasympathetic paragangliomas are usually located in the skull base and neck. Pheochromocytoma and paraganglioma are referred to as PPGL due to the similarity of histological features and physiological effects (2). Pheochromocytomas account for about 80% of PPGL and the rest are paragangliomas (3). The typical triad of PPGL is paroxysmal headache, palpitations, and heavy sweating. Other systemic signs and symptoms may also occur (4). Diagnosis of PPGL requires evidence of excessive CAs and localization of the tumor by imaging. Generally, the biochemical tests include CAs and intermediate metabolites of CAs in plasma or urine (5). In most cases, computed tomography (CT) should be the first choice for detecting tumors. Functional imaging, such as metaiodobenzylguanidine (MIBG) and somatostatin receptor(SSTR) scintigraphy, has advantages in detecting potential and metastatic lesions in PPGL (6). In 2017, the World Health Organization (WHO) replaced the previous concept of "malignant PPGL" with "metastatic PPGL" in the classification of neuroendocrine tumors. It is believed that all PPGLs have metastatic potential, and the concept of malignancy and benign was disregarded (7). In this paper, the complexity of PPGLs depends on the surgical difficulty. Therefore, PPGLs without surgical indications were excluded. The definition of complicated PPGLs in this paper is as follows: 1. The tumor is located in a complicated region (e.g. close to important blood vessels and organs), which can easily cause surgical collateral damage. 2. The tumor size is larger than 6cm with an obvious occupying effect (6, 8). 3. PPGL with unclear boundary with surrounding tissues and suspected invasion of adjacent tissues and organs. All the above conditions may significantly increase the difficulty of the operation. If any of the above conditions are met, it can be considered as complicated PPGLs.

## 2 Surgical region

**Figure 1:**
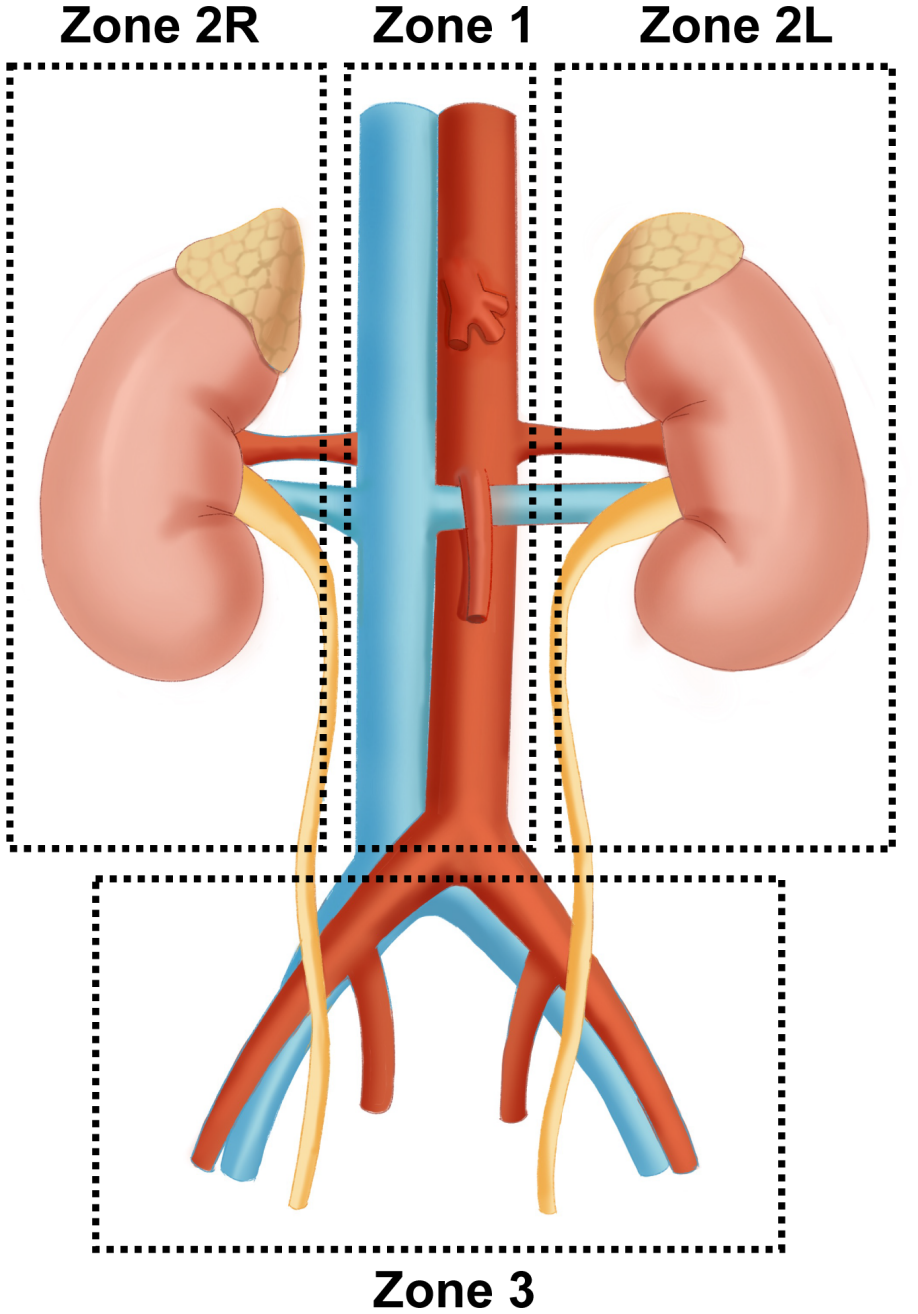
Surgical region of complicated PPGL Zone 1 (central vascular area) originates from the aortic hiatus and descends to the bifurcation of the iliac vessels, including the abdominal aorta, its main branches, and inferior vena cava. Zone 2 (Perirenal area) mainly contains bilateral kidneys and adrenal glands. Zone 3 (Pelvic area) mainly involves the bladder and its surrounding area (**Figure 1**).

The surgical treatment of PPGL is closely related to the anatomic location of paraganglia. Pheochromocytoma originates only from the adrenal medulla. In comparison, paraganglia is derived from embryonic neural crest cells and distributed in clusters throughout the body, mainly along the great vessels. Paraganglia is named for its location adjacent to the sympathetic and parasympathetic ganglia. Studies have shown that about 21% of paragangliomas are located in the abdominal cavity, which is often accompanied by the paraspinal sympathetic trunk and other less common sites, including the neck, chest, and bladder (9). Three operative areas were identified for a better surgical strategy according to important vascular routes such as the abdominal aorta, inferior vena cava, renal pedicle vessels, and iliac vessels: Zone 1 (central vascular area) originates from the aortic hiatus and descends to the bifurcation of the iliac vessels, including the abdominal aorta and the origin of its main branches (abdominal trunk, renal artery, superior/inferior mesenteric artery) and inferior vena cava. Zone 2 (Perirenal area) mainly contains the kidneys and adrenal glands. According to the different anatomical structures, Zone 2 is further divided into the left(L) and right(R) perirenal areas. First, adrenal venous reflux is different. The left adrenal central vein is longer and generally flows into the left renal vein. In comparison, the right one is concise and runs in front of the adrenal apex until it flows directly into the inferior vena cava (10). Besides, the left perirenal area is adjacent to the tail of the pancreas, spleen, and abdominal aorta, while the right perirenal area is adjacent to the duodenum, the head of the pancreas, the inferior vena cava, and portal vein. Clinical studies have shown that PPGL tends to occur in the perirenal area, especially near the hilum of the kidney. The tumors seem more likely to present on the left side, which may be related to the left-sided location of the abdominal aorta (11, 12). Zone 3 (Pelvic area) mainly involves the bladder and its surrounding area (**Figure 1**).

## 3 Surgical treatment strategy

After determining the qualitative and locational diagnosis of PPGL, the tumor should be surgically removed as soon as possible. Most PCCs are feasible for minimally invasive laparoscopic surgery, which has significant advantages in decreasing blood loss and pain, accurate dissection, shortening hospital stay, and reducing postoperative complications. Several surgical approaches are available for minimally invasive surgery. It mainly includes the transabdominal approach and retroperitoneal approach (13). The transabdominal approach provides the surgeon with a broader field of view and a larger operating space, and it is convenient to remove bilateral tumors. The transperitoneal lateral approach can achieve good therapeutic effects (14). The retroperitoneal approach has unique advantages in resecting the unilateral PCC at a shorter distance, which can effectively avoid abdominal organ injury (15). Both minimally invasive methods can be finished with robot assistance (16, 17). The Endocrine Society Guidelines recommended minimally invasive adrenalectomy (e.g. laparoscopic) for most pheochromocytomas(<6cm) (6). This cut-off was determined by several clinical studies that laparoscopic surgery for PCCs < 6cm means less intraoperative bleeding, shorter operating time, and less frequent hypertensive crises (18, 19). Although studies have shown that for experienced surgeons, laparoscopic adrenalectomy is superior to open adrenalectomy in reducing bleeding, hospitalization, and blood pressure management for selected PCCs larger than 6cm (20). However, as a risk factor affecting prognosis, tumor size (<6cm) is regarded as an important reference for surgical method selection (8). For large (e.g., >6 cm) or suspected invasive PCC, open surgery is recommended to ensure complete resection, prevent tumor rupture, and local recurrence (6).

The selection of surgical method and approach should be based on the principle of complete resection of the tumor. In addition to the standard open surgery, laparoscopic resection is feasible for small and non-invasive PGL by guidelines (6).

The choice of surgical approach for abdominal PGL mainly depends on the tumor location. In addition, since most of the supplying arteries of abdominal PGL originate from the abdominal aorta or its branches directly or indirectly, several factors such as tumor dissociation and vascular ligation also need to be considered (21). The retroperitoneal approach can also achieve good surgical efficacy for experienced surgeons (11, 22). And the safety and prognosis of retroperitoneal PGL with the retroperitoneoscopic and transperitoneoscopic approaches were proved no significant difference based on studies with large sample numbers (23). Therefore, surgeons can choose the most appropriate surgical procedure in combination with the guidelines, tumor conditions, and their own experience.

There are various factors contributing to the difficulty of PPGL surgery, which can be mainly attributed to the following points. First, the location of the tumor is complicated, such as the tumor surrounding the abdominal aorta and/or the inferior vena cava. There is a very high risk of vascular injury during surgical resection under these conditions. Second, PPGL can expand gradually with a size of more than 10cm in the retroperitoneal cavity. The occupying effect caused by the huge tumor and the unclear boundary with the surrounding tissue increases the surgical difficulty greatly. Therefore, PPGL surgery may involve multiple zones mentioned above, and we identified the zone with more than 50% tumor as the main surgical area for the convenience of summary. We share surgical treatment strategies based on several typical complicated PPGL cases.

### 3.1 PPGL in central vascular area

**Figure 2:**
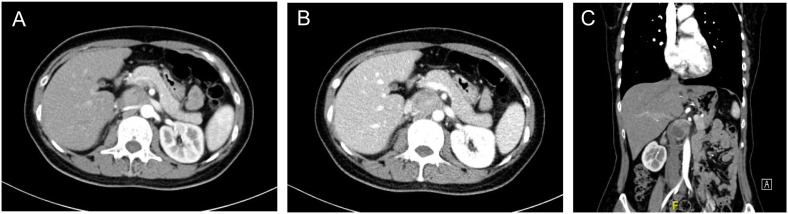
Enhanced CT of retroperitoneal PGL **(A)** Arterial phase of the PGL. The tumor was located behind the pancreas, pushing the right renal artery dorsally and adjacent to the abdominal aorta **(B)** Portal phase of the PGL. The inferior vena cava was pushed to the right and the left renal vein ran in front of the tumor. **(C)** Coronary position of the PGL.

Patient A is a 43-year-old female admitted for a 2-month paroxysmal headache, palpitations, and sweating. The diagnosis of abdominal para-aortic paraganglioma was considered by biochemical tests and radionuclide scintigraphy. Enhanced CT showed a 3.6cm×3.3cm retroperitoneal tumor with significant enhancement and unclear demarcation with the left renal vein, inferior vena cava, and abdominal aorta (**Figure 2**).

It is very challenging to manage PPGL embedded between the inferior vena cava and the abdominal aorta. First, the tumor was covered by the pancreas and pushed the inferior vena cava and the abdominal aorta laterally. The narrow space made both exposure and resection very troublesome. Second, the tumor was tightly restricted by the left renal vein and right renal artery, which increased the risk of intraoperative vascular injury. Besides, the pulsation of the abdominal aorta affects the tumor, leading to difficulty in the operation and higher requirements for precision. Finally, strong secretion of CA may cause hemodynamic instability.

The laparoscopic transabdominal approach was finally considered to dissociate precisely and reduce tumor irritation during operation. The lateral peritoneum was opened along the right paracolic sulcus. The ascending colon, duodenum, and the head of the pancreas were dissociated towards the midline to expose the tumor gradually. At the same time, assistants should carefully retract peritumor tissues and blood vessels to expand the operating space as much as possible. Since the tumor was located on the upper right side of the abdominal aorta, the ventral and vascularless sides of the tumor were explored at the beginning for simple dissociation. In addition, the tumor was adjacent to several important blood vessels and should be isolated along the surface carefully to avoid vascular injury. Gentle movement was necessary throughout the operation to reduce tumor irritation and avoid sharp fluctuations in blood pressure.

### 3.2 PCC in left perirenal area

**Figure 3:**
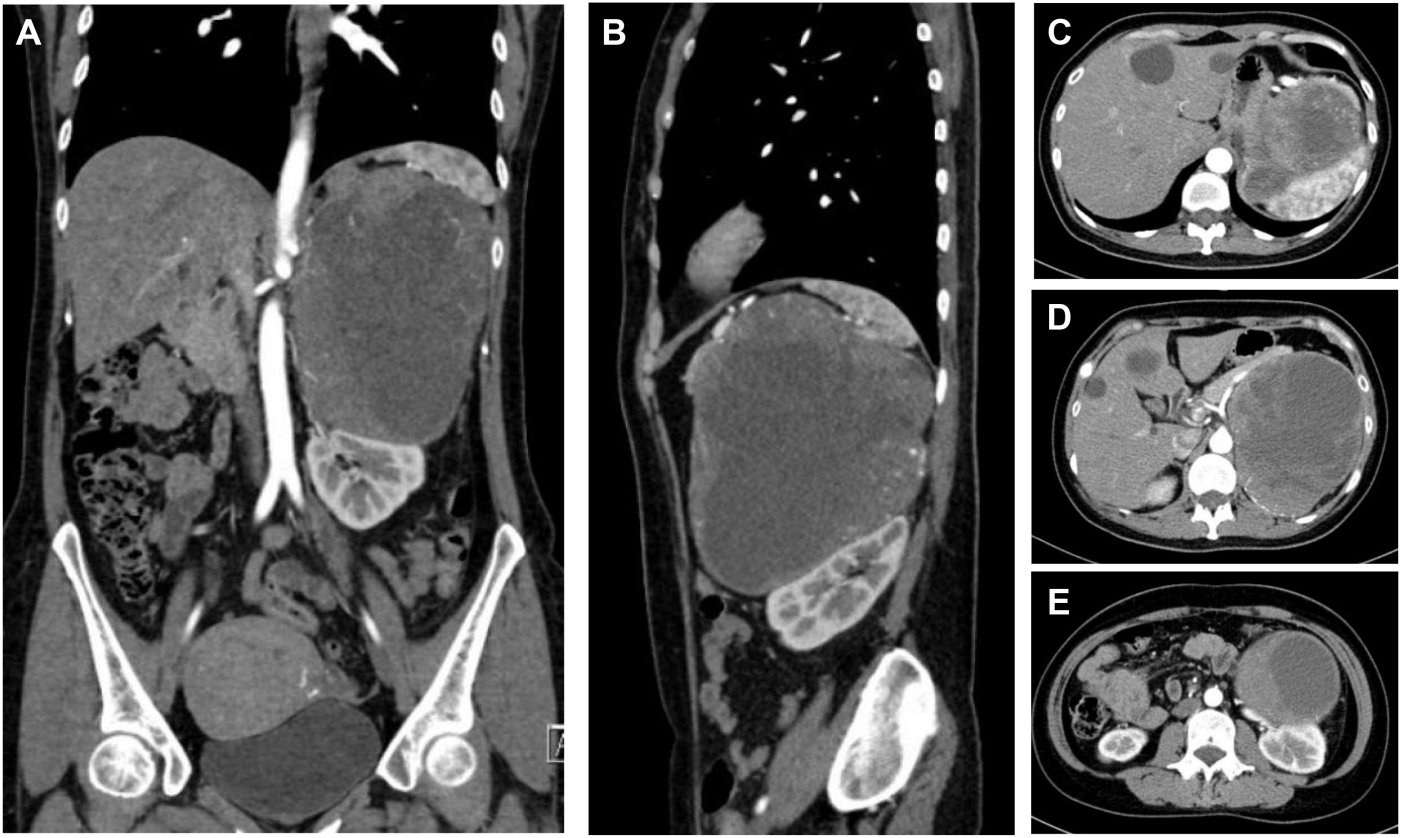
CT of a giant PCC in the left perirenal area. **(A)** Coronary position of the PCC. A mass of mixed density, with approximately 14.4cm×12.5cm in size, had significantly enhanced solid components. **(B)** Sagittal position of the PCC. The tumor pushed against the spleen and the left kidney. **(C)** The upper part of the PCC compressed the stomach. **(D)** The middle part of the PCC was not well demarcated with the tail of the pancreas and was close to the celiac trunk and the abdominal aorta. **(E)** The lower part of the PCC pressed on the left kidney and was close to the left renal pedicle.

Patient B was a 49-year-old female who suffered sudden palpitations and severe headaches with the highest blood pressure of 188/90 mmHg two months ago. The diagnosis of PCC was considered by biochemical tests and radionuclide scintigraphy. Enhanced CT showed a giant mass in the left upper abdomen with a cross-section size of 14.4cm×12.5cm. The low-density focus in the liver was hepatic cysts confirmed by enhanced CT (**Figure 3**).

The difficulty of this case was how to remove the entire tumor and avoid accidental injury. This giant tumor was mainly composed of cystic components with apparent occupying effects: the upper part of the tumor pressed against the spleen and stomach. The middle part was not demarcated from the tail of the pancreas and was close to the abdominal aorta and celiac trunk. The lower part pressed against the left kidney, which elongated the kidney pedicle significantly.

Generally, there is no fixed surgical approach for open surgery of massive retroperitoneal tumors, and the incision selection is usually based on total exposure and convenient operation. Open surgical treatment was performed through an arc incision 3cm below the costal margin to ensure complete resection. The tumor was fully exposed after the posterior peritoneum was opened through the left paracolic sulcus. The surface of giant PCC was coated with an envelope, which was separated from the surrounding tissue by a combination of blunt and sharp separation. The surgical plane should be accurately found, which could improve the dissociation efficiency and reduce bleeding. The cooperation of assistants was also essential. The tissue needed to be pulled away from the tumor as much as possible to expand the surgical space. The PCC was isolated from the opposite side of the abdominal aorta and left renal pedicle vessels to avoid injury. Tortuous vessels on the surface of the tumor were electrocoagulated, ligated, and dissected. Finally, the tumor was removed entirely after the blood supply vessels were tightly ligated with relatively stable hemodynamics.

### 3.3 PCC in right perirenal area

**Figure 4:**
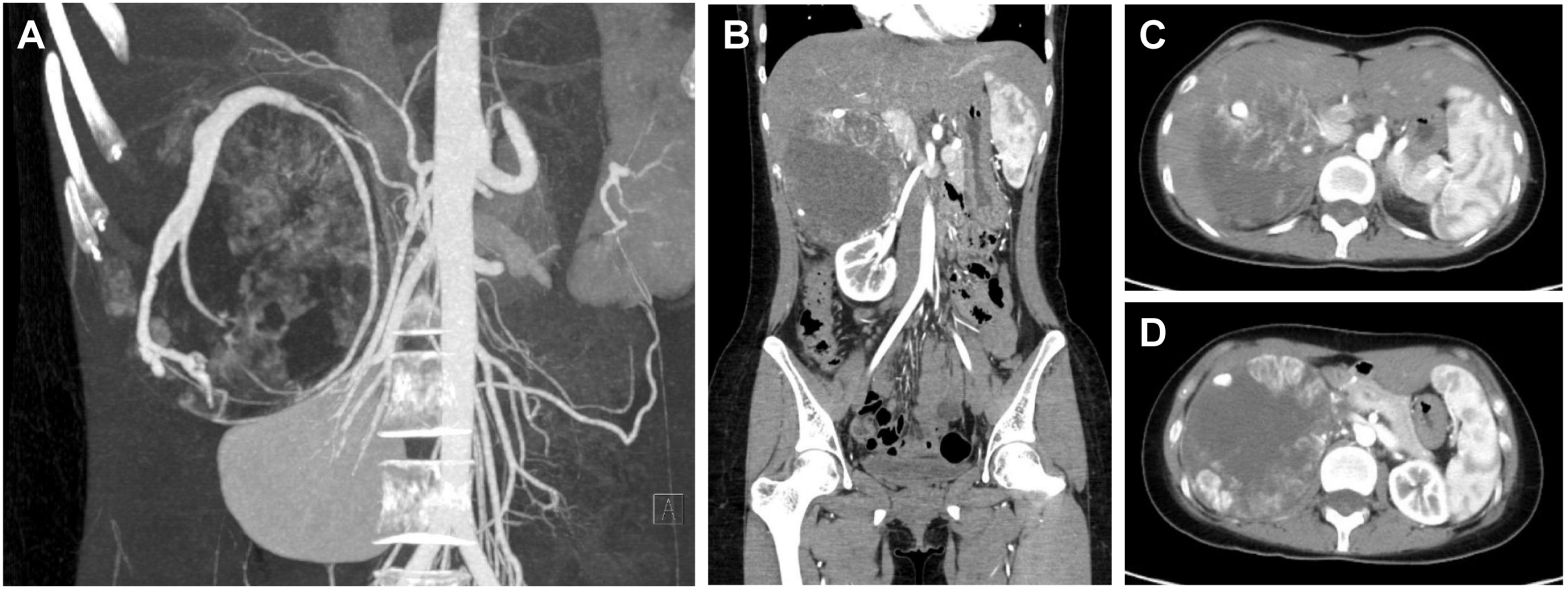
CT of a giant PCC in the right perirenal area. **(A)** CT of PCC angiography. Tortuous vessels with multiple aneurysmal-like dilatations were seen around the tumor. **(B)** Coronary position of the PCC. The tumor had a vague boundary with the liver and was close to the right renal pedicle. 10.9cm×9.0cm×10.7cm in size. **(C)** The upper part of the PCC was close to the inferior vena cava. **(D)** The maximum cross-section of the PCC was about 10.9cm×10.7cm.

Patient C was a 20-year-old female admitted for obvious palpitations, breath shortness, chest tightness, and dizziness during running for two years. The diagnosis of PCC was considered by biochemical tests and radionuclide scintigraphy. Enhanced CT showed a huge mass (10.9cm×9.0cm×10.7cm) in the right adrenal area, with uneven density and poorly defined boundary. There was a tortuous and thickened vessel in the tumor with multiple aneurysmal-like dilatations. It was considered the blood supply artery from the right adrenal artery (**Figure 4**).

The strategy of treating the right giant PCC is similar to the left one but still has its particularity. First, the right liver naturally occludes the PCC, so the surgical incision should be appropriately designed. Second, the space-occupying effect of the tumor pushed the pancreatic head, duodenum, inferior vena cava, and portal veins, resulting in a high risk of intraoperative organ and blood vessel injury. Besides, the malformed supply vessel ran longitudinally through the entire tumor, with multiple arteries originating from the abdominal aorta, which increased the difficulty of ligation and the potential risk of bleeding.

A “Y-shaped” incision was selected at the right side of the mid-abdomen for the convenience of exposure and dissociation. The right lobe of the liver was freed at the beginning. A giant PCC was shown after opening the posterior peritoneum through the right paracolic sulcus. Extra attention should be taken to avoid injury and severe complications near the pancreatic head and duodenum. Since the location of the blood supply artery was clear and shallow, it was ligated and cut off first. On the side near the abdominal aorta, all suspected small supply arteries were carefully ligated in case of postoperative hemorrhage. The inferior vena cava and portal vein were protected adequately during the operation due to the occupying effect of the considerable PCC. The tumor was finally removed with no extra injury.

### 3.4 PPGL in pelvic area

**Figure 5:**
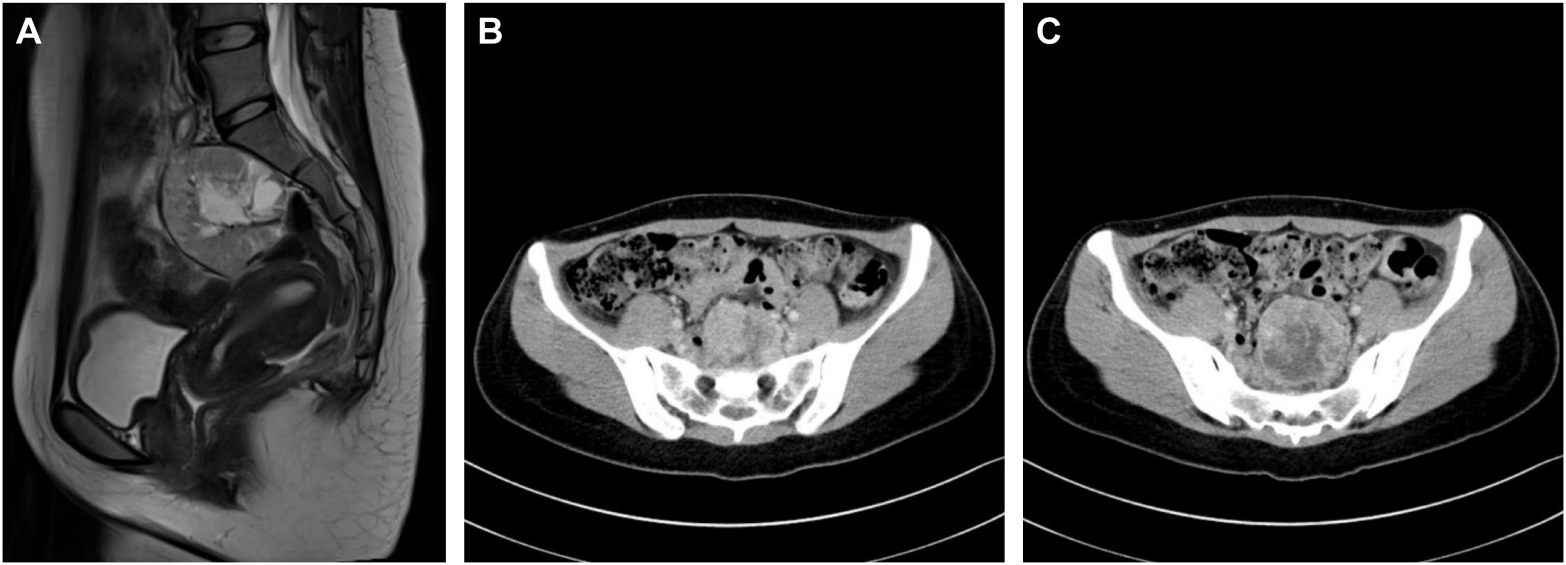
Imaging of a pelvic PGL **(A)** MRI of the sagittal position of the PGL. **(B)** The upper part of the PGL was close to the sacrum. **(C)** The maximum cross-section of the PCC was about 6.0cm×6.1cm.

Patient D, a 26-year-old female, was admitted due to hypertension, paroxysmal headache, and palpitations for 2 years. The diagnosis of PCC was considered by biochemical tests and radionuclide scintigraphy. CT and MRI identified a mass that was located above the uterus at the S1-S3 level, about 5.4cm×6.0cm×6.1cm in size, with uneven enhancement (**Figure 5**).

Surgical resection of pelvic PPGL is a challenge. The difficulty lies in that presacral tumors are often located deep, close to internal organs and blood vessels in the pelvic cavity. The narrow space of the pelvis will lead to an unclear surgical field and cause accidental injuries. Especially the presacral venous plexus bleeding will often cause relatively severe consequences (24). With several factors such as tumor size, location, and surgeon’s experience considered, the approach of transabdominal laparoscopic surgery was chosen with the Trendelenburg position. The tumor was exposed above the uterus and to the right of the sigmoid colon. First, the ventral side of the tumor was carefully dissociated. During the operation, the assistants needed to immobilize the tumor. Extra Trocar should be available if necessary. The surgeon was supposed to carefully separate the plane between the tumor surface and the sacrum to reduce irritation and accidental injury. The endoscopic gauze was packed appropriately to protect the presacral venous plexus. The action should be gentle and avoid scratching. The presacral venous plexus should be carefully checked before the end of the procedure in case of hemorrhage after closing the pneumoperitoneum.

## 4 Multidisciplinary treatment of PPGL

Pheochromocytoma/paraganglioma are neuroendocrine-related tumors with varied clinical symptoms and pathophysiological features due to the excessive secretion of CAs. Multidisciplinary treatment (MDT) has gradually become a routine process for PPGL because of the complexity of preoperative diagnosis, treatment, and postoperative care. After integrating the various opinions, the MDT model can develop the best strategies for patients, significantly improving the efficiency and quality of treatment (25, 26).

The Endocrine Society Guidelines indicated that imaging examination should be performed in potential PPGL patients with positive biochemical evidence. CT is considered the preferred method for its excellent spatial resolution in conventional imaging (6). On unenhanced CT, PPGL could show various appearances. The density of PPGL may be homogeneous or heterogeneous. About two-thirds of PPGLs are solid with soft-tissue density, and the rest are cystic or solid-cystic (27). As a neuroendocrine tumor associated with a genetic background, there were some significant differences between hereditary and sporadic PCC. Chung et al found that sporadic PCCs were larger in size with more cystic or necrotic components than the hereditary ones (28). Enhanced CT has a higher sensitivity for locating PPGL as small as about 5 mm. Almost 100% PPGLs showed a mean attenuation of more than 10 Hounsfield units (HU) and more than 60% washout in a 15-minute delay (29). MRI was applied in certain populations such as children, pregnant women, and patients who have recently been overexposed to radiation or are allergic to contrast (6). Relatively high signal intensity on T2-weighted images was shown in most PPGLs (30).

Since the definition of potential metastasis risk of PPGL was confirmed in 2017, molecular imaging has played an important role in diagnosis. Compared with conventional CT/MRI, positron emission tomography (PET)/CT with radiopharmaceuticals has more advantages in evaluating metastatic/multifocal PPGL and adjacent organ infiltration. ^18^F-fluorodeoxyglucose (^18^F-FDG) is one of the most widely used PET/CT markers in clinics. An amount of glucose was needed by tumor tissues due to the rapid growth and anaerobic metabolism, and ^18^F-FDG is transported into the cell but does not participate in metabolism, and eventually marks tumor tissues (31). A clinical study involving 216 patients with PPGL compared the sensitivity and specificity of ^18^F-FDG-PET/CT with CT, MRI, and ^123^I-MIBG. The results showed that the sensitivity of ^18^F-FDG-PET/CT was similar to that of ^123^I-MIBG (76.8% vs 75.0%) and its specificity was similar to that of ^123^I-MIBG and CT/MRI for non-metastatic tumors (90.2% vs 91.8% vs 90.2%). For metastatic PPGL, the sensitivity of ^18^F-FDG-PET/CT was significantly higher than that of ^123^I-MIBG and CT/MRI (82.5% vs 50.0% vs 74.4%) (32). In recent years, ^68^Ga-DOTA (0)-Tyr (3)-octreotate (^68^Ga-DOTATATE) PET/CT has attracted more attention. Several clinical researches reported that the lesion detection sensitivity for ^68^Ga-DOTATATE PET/CT was significantly higher than that of ^18^F-FDG PET/CT, CT/MR, and ^131^I-MIBG scintigraphy especially in pediatric PPGL cohort (33, 34). The application of ^68^Ga-DOTATATE was approved by the US Food and Drug Administration (33).

Due to the complexity of PPGL, it is often tricky for the department of urology to manage alone. For example, vascular surgery strongly supports vessel dissociation, ligation, repairment, and thrombotic events management (35). Hepatic surgery supports liver dissociation and portal vessel protection (36). General surgery provides essential help in abdominal organ dissociation and multiple organ resection (37). Because of the MDT system, surgical protocols are made to fulfill individual needs, which eventually benefits patients.

PPGL is a kind of tumor with metastatic potential, and there is a risk of local or metastatic recurrence even after the tumor is completely removed. Therefore, it is necessary to maintain postoperative follow-up and surveillance (25). The test of intermediate metabolites of catecholamine — metanephrine(MN) and normetanephrine(NMN) in plasma has been widely used in clinical with good sensitivity and specificity (38, 39). For the short-term postoperative follow-up, the monitoring of MN and NMN was recommended after recovery from surgery in 2–6 weeks. All the postoperative patients with PPGLs are supposed to keep at least a 10-year follow-up to monitor recurrences or new tumors, especially with High-risk factors (young ages, heredity, large size, and/or a paraganglioma) (40). In general, annual routine physical examinations and clinical follow-ups are recommended for PPGL patients. Imaging examinations are necessary among suspected cases. Functional imaging (e.g.^123^I-MIBG scintigraphy) has an advantage in diagnosing metastasis or recurrence. ^18^ F-FDG PET/CT is suitable for definite metastatic cases (6). For patients who have developed metastases or whose tumors cannot be completely removed by surgery, although the efficacy of systematic treatment is limited, it can still relieve symptoms and control the disease to a certain extent. Individualized therapy plans require multidisciplinary collaboration (41).

In addition to the surgical department, several other departments play an essential role in PPGL-MDT. The endocrinology department plays a vital role in regulating blood pressure and managing PPGL-related complications, dramatically improving patients’ quality of life, and laying the foundation for surgery. Excessive CAs secreted by PPGL may cause a hypertensive crisis, low blood volume, arrhythmias, myocardial ischemia, pulmonary edema, stroke et al. (42). Before medical treatment became widespread in the 1950s, the risk of perioperative death in adults was nearly 45%. This rate could be reduced to less than 2% via systematic preoperative blood pressure monitoring by medicine therapy (α-adrenergic antagonists, calcium channel blockers, and β-receptor blockers) (25, 43).

α-adrenergic receptor (AR) antagonists are recommended by the guidelines for PPGL complications. Phenoxybenzamine is a non-selective AR antagonist commonly used for perioperative blood pressure control. In general, phenoxybenzamine should be initiated at least 7-14 days before surgery to ensure adequate blocking of α-receptors. The initial dose of phenoxybenzamine is 10mg twice a day, which can be increased or decreased depending on symptoms until the patient has normal blood pressure and no sudden tachycardia. The maximum daily dose can reach 1mg/kg (42). According to our clinical experience, for complicated PPGLs or PPGLs with the strong secretion of catecholamine, an appropriate extension of phenoxybenzamine administration time can better control PPGL symptoms. We generally extend the duration of phenoxybenzamine to at least 1 month. Some side effects of phenoxybenzamine can also be indicators of preoperative preparation besides blood pressure. In general, the blood pressure of PPGL patients with adequate medication preparation is generally less than 140/90 mmHg. Patients may have mild postural hypotension or nasal congestion. In addition, the antagonistic effect of phenoxybenzamine on α receptors will cause vasodilation and increase blood volume. Hematocrit is generally less than 40%, and the patient will gain weight and the fingertips become warm from cold.

Selective AR antagonists such as doxazosin can also achieve ideal clinical efficacy. Studies have shown that there is no statistical difference in efficacy between selective AR antagonists and non-selective AR antagonists (44, 45). When hypertension is not well controlled by α-AR antagonists alone, calcium channel blockers (CCBs) are often combined to further improve blood pressure control in PPGL patients. CCBs inhibit the norepinephrine-induced transmembrane calcium influx of vascular smooth muscle, thereby reducing peripheral vascular resistance and blood pressure (46). Multiple studies have demonstrated that CCBs have similar effects to α-AR antagonists on hemodynamic stability during the perioperative period (47, 48). PPGL-related tachycardia can be effectively controlled by β-AR antagonists (6). It is noticeable that β-AR antagonists are contraindicated in patients with PPGL alone or before α-AR is adequately treated. Because β-AR antagonists may cause loss of vasodilation before α-AR is adequately blocked, resulting in a sharp increase in blood pressure, and even hypertensive crisis (49).

Secondary hypertension and hemodynamic instability are common complications of PPGL. Patients with excessive CA secretion of PPGL often have a paroxysmal headache, secondary hypertension, large blood pressure fluctuation, and severe postural hypotension. In clinical practice, blood pressure is an important indicator to reflect PPGL patients’ medical preparation and physical conditions and it is generally considered that patients with PPGL should control their blood pressure at 140/90 mmHg, and the ideal value is 120/80 mmHg without obvious postural hypotension (46). Therefore, ambulatory blood pressure monitoring (ABPM) is an important method of evaluation. Increasing studies identified that ABPM plays an indispensable role in the routine of secondary hypertension, which revealed the blood pressure pattern of PPGLs (50, 51). Although 24h-ABPM is not currently a routine blood pressure monitoring method in our hospital, its clinical value is widely recognized. Therefore, the anesthesiologist will pay attention to the fluctuation of patients’ blood pressure and the conditions of vital organs during the intraoperative period of high risk, such as endotracheal intubation, operation on a tumor, and ligation of essential blood vessels (52). In our hospital, a PPGL-related group of anesthesiologists has been established to have a complete and detailed pre-anesthetic evaluation. Under the guidance of experienced anesthesiologists, personalized anesthesia protocols are made based on the surgical procedure and physical conditions, which makes the surgical procedures run smoothly.

The ICU provides a solid guarantee for the postoperative treatment of PPGL. Some studies have shown that ICU monitoring is unnecessary when the patient is hemodynamically stable during surgery (46). However, postoperative complications should not be underestimated. The most common is persistent hypotension due to loss of blood, impaired vascular compliance, or residual effects of the preoperative adrenergic blockade. Other complications, such as hypertensive crisis, severe arrhythmia, and hemorrhagic shock, could endanger life in severe conditions (53). Therefore, patients with the risk for potential hemodynamic instability still need to have closely cared in the ICU during the first 24-48 hours after surgery (54). In combination with our clinical experience, all these complicated PPGL cases were transferred to the ICU due to the long-time operation and high risk of postoperative hemodynamic instability. They were transferred back 24 or 48 hours later with vital signs in the normal range.

According to Toniato et al, PPGL surgery is not more difficult than the laparoscopic adrenalectomy for incidentaloma, Conn’s disease, and Cushing’s disease (55). So surgical treatment of common PPGL is feasible for most hospitals. However, in complicated PPGL cases (e.g., large tumor size, tumor compressing important blood vessels or organs, PPGL-induced hemodynamic instability), experienced surgeons and anesthesiologists, proper perioperative monitoring, and a well-cooperated multidisciplinary team can maximize the safety and postoperative recovery of patients. Therefore, complicated PPGL patients should be treated in general hospitals with specialized PPGL surgical teams.

In conclusion, PPGL has diverse clinical manifestations and complex complications, which may involve multiple organs and systems. The MDT-PPGL model can effectively evaluate the tumor, formulate the treatment plan, and complete the postoperative monitoring, which fully guarantees efficacy and safety. We will continue to summarize the surgical management strategy of PPGL in future clinical work so that more patients can benefit from it.

## Data availability statement

The raw data supporting the conclusions of this article will be made available by the authors, without undue reservation.

## Ethics statement

The studies involving human participants were reviewed and approved by Ethic committee of Peking Union Medical College Hospital. The patients/participants provided their written informed consent to participate in this study.

## Author contributions

YSZ led the operation team. XW, YZ and ZL took part in the operation. XW acquired the data and prepared the first draft. YSZ, YZ and ZL reviewed critically and contributed to the final revision. All authors read and approved the final manuscript. All authors contributed to the article and approved the submitted version.

## Supplementary material

Supplementary Figure 1 Intraoperative gross images of the PPGLs A–D represents the gross PPGL of patients **(A–D)** respectively.
