# Supplementary figures and images for: Surgical strategies of complicated pheochromocytomas/paragangliomas and literature review
Source: Front Endocrinol (Lausanne). 2023 Apr 21;14:1129622. doi: 10.3389/fendo.2023.1129622 (PMC10160616; doi:10.3389/fendo.2023.1129622)

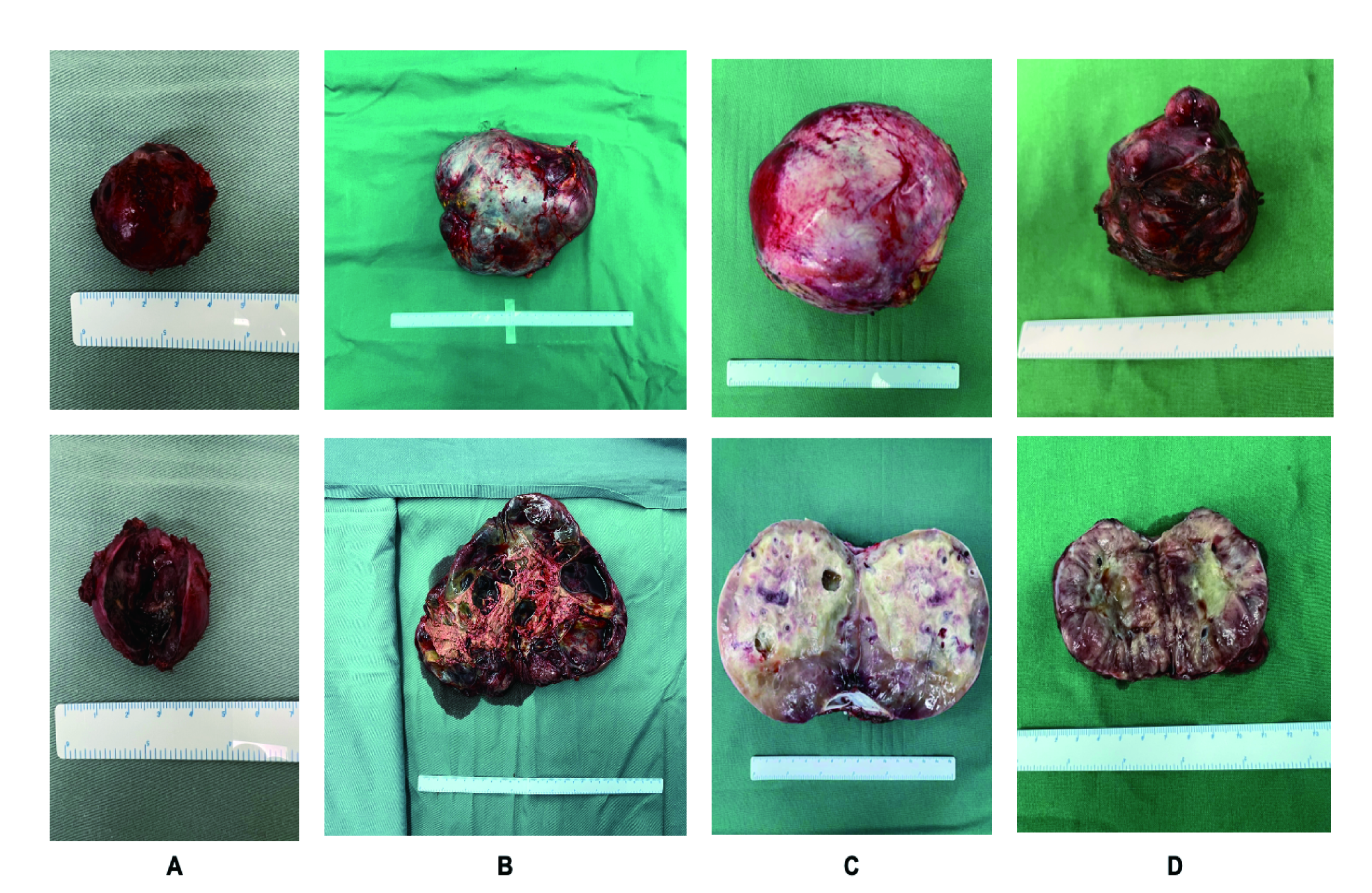

Supplement: Supplementary Figure 1 — Intraoperative gross images of the PPGLs A–D represents the gross PPGL of patients (A–D) respectively. [file Image_1.tif]
